# Transversions have larger regulatory effects than transitions

**DOI:** 10.1186/s12864-017-3785-4

**Published:** 2017-05-19

**Authors:** Cong Guo, Ian C. McDowell, Michael Nodzenski, Denise M. Scholtens, Andrew S. Allen, William L. Lowe, Timothy E. Reddy

**Affiliations:** 1 Center for Genomic and Computational Biology, Duke University Medical School, Durham, NC 27710 USA; 2 University Program in Genetics and Genomics, Duke University, Durham, NC 27710 USA; 3 Program in Computational Biology and Bioinformatics, Duke University, Durham, NC 27710 USA; 4 Department of Preventive Medicine, Division of Biostatistics, Northwestern University Feinberg School of Medicine, Chicago, IL 60611 USA; 5 Center for Statistical Genetics and Genomics, Duke University Durham, North Carolina, 27710 USA; 6 Department of Biostatistics and Bioinformatics, Duke University Medical School, Durham, NC 27710 USA; 7 Division of Endocrinology, Metabolism and Molecular Medicine, Department of Medicine, Northwestern University Feinberg School of Medicine, Chicago, IL 60611 USA; 8 Present Address: Biostatistics & Bioinformatics, 101 Science Dr., 2347 CIEMAS, Durham, NC 27708 USA

**Keywords:** Transitions, Transversions, Massively parallel reporter assay, SNPs, Regulatory variation

## Abstract

**Background:**

Transversions (Tv’s) are more likely to alter the amino acid sequence of proteins than transitions (Ts’s), and local deviations in the Ts:Tv ratio are indicative of evolutionary selection on genes. Whether the two different types of mutations have different effects in non-protein-coding sequences remains unknown. Genetic variants primarily impact gene expression by disrupting the binding of transcription factors (TFs) and other DNA-binding proteins. Because Tv’s cause larger changes in the shape of a DNA backbone, we hypothesized that Tv’s would have larger impacts on TF binding and gene expression.

**Results:**

Here, we provide multiple lines of evidence demonstrating that Tv’s have larger impacts on regulatory DNA including analyses of TF binding motifs and allele-specific TF binding. In these analyses, we observed a depletion of Tv’s within TF binding motifs and TF binding sites. Using massively parallel population-scale reporter assays, we also provided empirical evidence that Tv’s have larger effects than Ts’s on the activity of human gene regulatory elements.

**Conclusions:**

Tv’s are more likely to disrupt TF binding, resulting in larger changes in gene expression. Although the observed differences are small, these findings represent a novel, fundamental property of regulatory variation. Understanding the features of functional non-coding variation could be valuable for revealing the genetic underpinnings of complex traits and diseases in future studies.

**Electronic supplementary material:**

The online version of this article (doi:10.1186/s12864-017-3785-4) contains supplementary material, which is available to authorized users.

## Background

There are millions of candidate gene regulatory elements across diverse human cell types, tissues, and environmental conditions (e.g. [1–4]). Genetic variation in those candidate regulatory elements contributes heavily to the variation in gene expression between individuals and, in turn, to the heritability of complex human traits and diseases [5–7]. Determining the specific genetic contributions to both molecular and organismal traits remains a major challenge, however. That challenge persists, in part, because it is difficult to predict the effect that a given variant or set of variants is likely to have on gene regulation. Overcoming that challenge is important for both basic and translational studies of the genetics of gene regulation [8, 9].

A wide variety of studies have now investigated the genetic contributions to human gene expression. Studies of the associations between genotype with gene expression has revealed genetic contributions to nearly every human gene and across diverse cell types and tissues [10]. Meanwhile, studies of the allele-specific binding of transcription factors (TFs) suggest that noncoding variants can alter gene regulation by several mechanisms: disrupting TF binding directly, disrupting complexes of regulatory factors, and disrupting the underlying chromatin state [11–14]. A challenge the above studies face is that genotypes near each other in the genome are highly correlated due predominantly to limited and non-random sites of meiotic recombination across the human genome (i.e. linkage disequilibrium). As one solution to that challenge, investigators have used reporter gene expression assays to measure the effects of genetic variation on the activity of regulatory elements across the genome [15, 16]. In a standard reporter gene expression assay, a regulatory element drives expression of a visually observable reporter gene such as a fluorescent or chemiluminescent protein. By assaying regulatory elements with different genotypes, it is possible to identify genetic variants that directly alter the activity of those elements. Recently, high-throughput versions of those assays have been developed to measure the regulatory effects of many genetic variants and mutants at once [17–20]. In such assays, the regulatory elements drive expression of DNA-encoded barcodes that allow for readout with high-throughput sequencing.

While there are many ways to investigate how genetic variants influence gene regulation, performing those studies in the primary cells and tissues that are most relevant to organismal biology remains challenging. For that reason, understanding which variants to prioritize for testing will be highly valuable. More generally, determining which types of mutations are most likely to influence gene regulation will also be important for studying role of regulatory variation in evolution. As a step towards that long-term goal, we focused on testing whether there are effect differences between the two types of genetic mutations, transitions (Ts’s) and tranversions (Tv’s). Transitions are DNA mutations that maintain the same number of rings in the nucleotide base, specifically exchanging a one-ring pyrimidine with another pyrimidine, or a two-ring purine for another purine. Transversions, in contrast, are mutations that change the nucleotide base from a purine to a pyrimidine or vice versa. It is well known that Ts’s are enriched over Tv’s in protein-coding regions of the human genome. One of the reasons that Tv’s are thought to be depleted in exons is that they are more likely to result in an amino acid substitution. That difference between the rate of Ts’s and Tv’s is a foundational principle for studies of the molecular basis for evolution [21–23]. In contrast, the different effects that Ts’s and Tv’s have in the non-coding genome has not been as well studied. One of the major ways the genetic mutations alter regulatory element activity is by influencing the affinity of TFs to the genome [15, 16, 24]. TFs bind DNA based on both sequence and shape [25]. Here, we show that Tv’s are more likely than Ts’s to alter local DNA structure, TF binding and, in turn, regulatory element activity. To do so, we integrated data from numerous orthogonal studies of the genetic effects on DNA structure, TF binding, and regulatory element activity. While much remains to be understood, our findings enhance understanding of the effects of genetic variation on human gene regulation.

## Results

### Tv’s alter DNA minor groove width and roll more than Ts’s

**Fig. 1:**
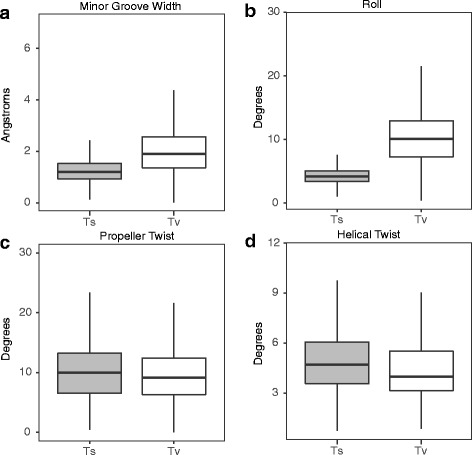
Tv’s have greater effects on DNA shape. Each plot shows the estimated effect of Ts’s and Tv’s placed in the center of random 500 bp nucleotide sequences on (**a**) minor groove width, (**b**) roll, (**c**), propeller twist, and (**d**) helical twist, as estimated by DNAshapeR. Each plot shows data for all four possible variants of 100,000 random sequences, for a total of 400,000 random sequences analyzed

We first hypothesized that Tv’s have a greater impact on the shape of DNA than Ts’s. We tested that hypothesis using an empirically-based model of the effect of DNA sequence on DNA shape [26] that has been used previously to investigate the shape readout of TFs [27]. We used that model to predict the effect of Tv’s and Ts’s embedded in the center of 501 bp DNA sequences on four DNA shape parameters: minor groove width (MGW), propeller twist (ProT), helical twist (HelT), and roll (Fig. 1). Transversions had substantially greater effects on minor grove width (2 Å vs 1.3 Å, an increase of 1.5×) and on roll (10.2° vs 4.4°, an increase of 2.3×). In contrast, the Ts’s had greater effects than Tv’s on HelT and ProT, but the magnitude of the effects was much smaller (1.09× and 1.14×, respectively). Overall, these results indicate that Tv’s overall have a greater impact than Ts’s on DNA shape, and disproportionately alter the minor groove width and roll of DNA.

### Tv’s have greater impacts on predicted and experimentally-measured TF binding

**Fig. 2:**
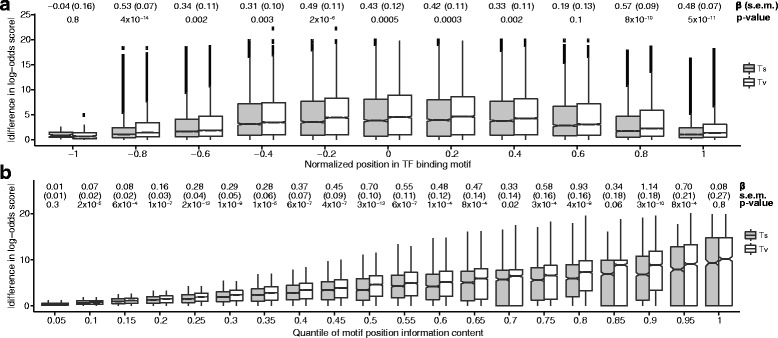
Tv’s are depleted at TF binding sites. **a**, **b** Changes to PWM scores for JASPAR TFs caused by Ts’s (*grey boxplots*) and Tv’s (*white boxplots*) ordered by normalized position (*above*) and information content (*below*). For each position in each consensus sequence, we calculated the position specific scoring matrix (PSSM) score of having every possible nucleotide at that position and, subsequently, the change in PSSM score when mutating any nucleotide to any other nucleotide at that position. *Black* squares are changes >1.5× above the interquartile range. *P*-values for parameter estimates were calculated using a t test, and are reported without adjustment for multiple hypothesis testing. We tested 11 and 20 hypotheses in Fig. 2a and b, respectively. Applying a Bonferroni correction to a nominal *p*-value threshold of α = 0.05 gives a significance threshold of α = 0.005 and α = 0.0025 for 2A and 2B, respectively

We next hypothesized that Tv’s also have greater effects on TF binding than Ts’s. We first evaluated whether Tv’s have a greater predicted effect according to computational and statistical models of the TF:DNA interaction. To do so, we calculated the change in the position weight matrix (PWM) score of every possible single nucleotide mutation in every TF binding motif in the JASPAR database [28]. Briefly, a PWM quantifies the affinity of a TF to each nucleotide in a potential binding site. The center of the TF binding motif is typically more specific than either edge of the motif and, similarly, mutations near the center of the motif typically had greater impacts on the PWM score. Across all motifs, Tv’s had a significantly greater effect on TF binding score both in the center of the motif and in the flanking regions (Fig. 2a). The effect was most pronounced at motif positions with moderate nucleotide specificity (i.e. information content [29]), suggesting that degeneracy in TF binding motifs more often accommodate Ts’s than Tv’s (Fig. 2b). Together, these results indicate that, across all TFs, Tv’s are predicted to have a greater impact on TF binding than Ts’s.

**Fig. 3:**
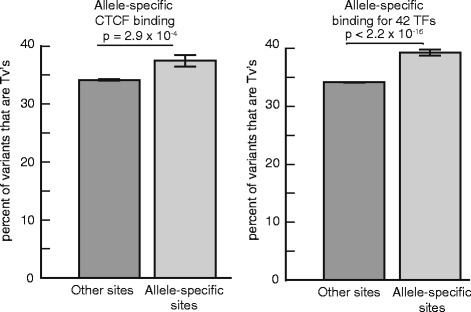
Tv’s are enriched in allele-specific transcription factor binding sites across the genome. The percentage of Tv’s in allele-specific CTCF across six LCL lines (*left*), and for allele-specific binding across seven TFs (*right*) in a publically available database [30]. *P*-values and parameter estimates were calculated using a two-tailed Z-test. Error bars show the s.e.m

To test whether the computational predictions are realized in the genome, we next investigated whether there were also greater differences in TF binding between alleles at Tv’s than at Ts’s. To do so, we analyzed publicly available allele-specific ChIP-seq data for 42 TFs in the fully-sequenced diploid lymphoblastoid cell line (LCL) GM12878, and for CTCF across six LCLs [30]. In both instances, SNPs with evidence of allele-specific TF binding were subtly but significantly enriched for Tv’s when compared to the other SNPs tested (39.33% vs 34.16% for TFs in GM12878 cells, 37.4% vs 34.16% for CTCF in LCLs; Z test *p* < 2.2 ×- 10^−16^, 2.86 × 10^−4^, respectively, Fig. 3). These results suggest that Tv’s have larger effects on TF binding, resulting in their depletion within TF binding motifs and sites.

### Tv’s have greater impacts on functional regulatory element activity

Based on our results showing that Tv’s have greater effects than Ts’s on TF binding, we next hypothesized that Tv’s would also have greater effects on regulatory element activity. Because non-coding variation is expected to be a major contribution to human traits and diseases, we focused our analysis on variants on a region on chromosome 3 in which we previously found genetic variants associated with birth weight and fetal adiposity [31–33]. We chose that region as a representative example of a region of the human genome that is associated with a complex human trait or disease. Within that region, we focused specifically on 104 regions that are hypersensitive to digestion by DNaseI (i.e. DNase hypersensitive sites, or DHS’s) (Additional file 1: Figure S1, Additional file 2: Table S1). DHS are strongly indicative of TF binding and regulatory element activity, and are also strongly enriched for gene regulatory SNPs associated with human traits [34–36]. By focusing our experiments on DHS at a trait associated locus within a population, we increased our likelihood of capturing expression modulating variants that are relevant to a human phenotype.

To measure the regulatory activity of diverse haplotypes of the 104 DHSs, we used a high-throughput population-scale self-transcribing active regulatory region sequencing (STARR-seq) reporter assay that we call POP-STARR [37]. Briefly, in POP-STARR, candidate regulatory elements from a population of individuals are cloned into the 3′ untranslated region (UTR) of the STARR-seq reporter gene [38]. From that position, each regulatory element control expression of a reporter gene in which it is embedded. For example, once the library is transfected into cells, the regulatory elements with a high level of activity are found frequently in the pool of expressed reporter gene mRNA relative to the regulatory elements with low activity. One can then measure the abundance, and therefore activity, of all regulatory elements in the library by using massively parallel DNA sequencing. Importantly, genotype is observed by DNA sequencing as well. The result of that measurement, therefore, is an allele-specific measure of regulatory element activity across the captured regulatory elements and across the population of from which the regulatory elements were captured.

**Fig. 4:**
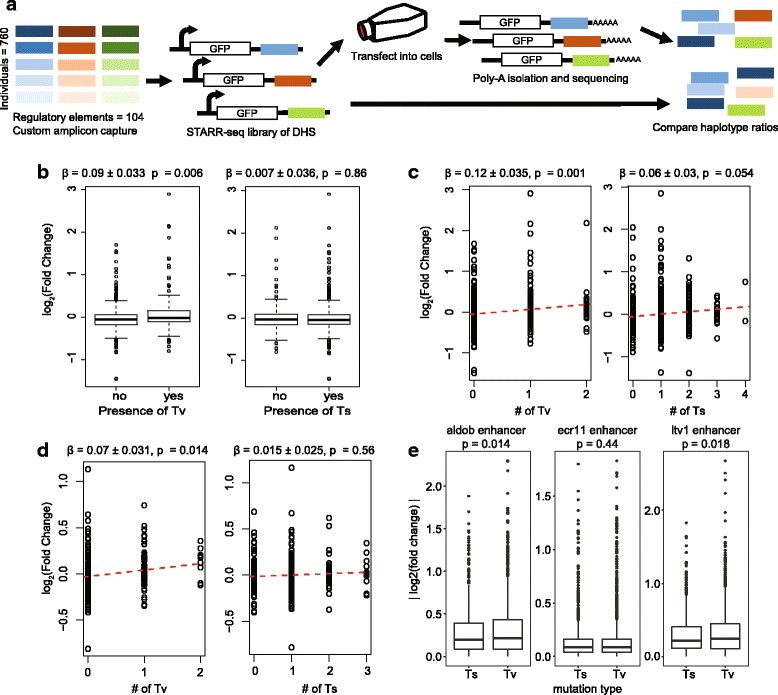
Tv’s have greater regulatory effects than Ts’s. **a** Schematic of POP-STARR experimental design. **b** Changes in haplotype effect magnitudes due to the presence of a Ts or Tv within a haplotype. *Black squares* are effects 1.5× of the interquartile range above the upper quartile or below the lower quartile. *P*-values were calculated using a t test. **c** Correlation between change in haplotype effect magnitude and the number of Ts/Tv differences between haplotypes. The *red line* is the regression line. *P*-values were calculated using a t test. **d** Correlation between change in haplotype effect magnitude and the number of Ts/Tv differences between haplotypes which overlap DHS centers. The red line is the regression line. *P*-values were calculated using a t test. **e** *Boxplots* of the effect of transitions and transversions on regulatory element activity as measured in Patwardhan et al., [19]. Each plot shows data from a different enhancer. Each plot shows the absolute log_2_(fold change) in regulatory element activity for every Ts and Tv assayed in that enhancer. *P*-values are for a one-sided t test from a linear regression model

To test our hypothesis that Tv’s have a greater effect on regulatory element activity than Ts’s, we use POP-STARR to assay the activity of 104 DHSs captured from the genomes of 760 donors (Fig. 4a). In total, we assayed 1153 unique haplotypes comprised of 942 variants. Of those variants, 634 were Ts’s and 308 were Tv’s (Additional files 3 and 4: Tables S5-S6). To then test if Tv’s alter the activity of regulatory elements more than Ts’s, we classified haplotypes by whether they contained a Tv or a Ts relative to a reference haplotype. We then used a multiple linear regression model to test if the presence or absence of a Tv or Ts correlated with changes in regulatory activity between haplotypes. The presence of a Tv was correlated with greater changes in regulatory activity (t test, β = 0.09 ± 0.033 s.e.m., *p* = 0.006), while the presence a Ts was not (t test, β = 0.007 ± 0.036 s.e.m., *p* = 0.86) (Fig. 4b). This observation suggests that haplotypes within regulatory elements that contain Tv’s are more likely to impact activity. Next, we expanded the model to account for the total number of Ts’s and Tv’s between haplotypes. The total number of Tv’s was significantly correlated with the magnitude of changes in regulatory activity (t test, *p* = 0.001) whereas the total number of Ts’s was not (t test, *p* = 0.054). Furthermore, the effect of additional Tv’s on the magnitude of changes in regulatory element activity was double that of additional Ts’s (β = 0.12 ± 0.035 s.e.m vs 0.06 ± 0.03 s.e.m.) (Fig. 4c). Since TF binding is enriched at the center of DHS’s, we hypothesized that that relative magnitude of effect between Tv’s and Ts’s would increase near the middle of DHS’s. When the same analysis was limited to haplotypes that overlapped the middle third of DHS’s, the effect of Tv’s was substantially larger (β = 0.07 ± 0.031 s.e.m.), the effect of Ts’s was unchanged (β = 0.015 ± 0.025 s.e.m.), and the ratio of the effect sizes increased to 4.6-fold (Fig. 4d).

To confirm that our results are not specific to our model system or the 3q25 locus, we performed a similar analysis on a study that used saturation mutagenesis to evaluate the effect of every possible mutation on the activity of three enhancers [19]. In that study, Patwardhan et al. used massively parallel reporter assays to measure the effect of every possible single nucleotide change to three known enhancer regions. The saturation mutagenesis approach allowed for quantification of the effects of every possible mutation on regulatory activity. As in our POP-STARR results, Tv’s had greater effects on activity than Ts’s, and that difference was greater near the center of each regulatory element (Fig. 4e, Additional file 1: Table S7). Together, these results confirm the results of our POP-STARR assays in an alternative high-throughput reporter system, providing further empirical evidence that Tv’s have larger impacts on regulatory element activity than Ts’s for both naturally occurring variants and artificially generated mutations.

## Discussion

Although the impacts of Ts’s and Tv’s have been extensively studied in coding sequences, differences in their effects in non-coding DNA has remained largely overlooked. Here, we have shown that there are functional differences in the effects of Ts’s and Tv’s in non-coding regulatory elements. Specifically, our results show that Tv’s are more likely to alter DNA shape, to disrupt TF binding, and to have larger effects on regulatory element activity than Ts’s. These findings represent a novel, fundamental property of regulatory variation.

The observed overall effects of Tv’s and Ts’s on regulatory element activity are modest. That finding is as expected considering earlier results showing that most genetic mutations or variants have modest effects on regulatory element activity [16, 19, 37]. Our estimates of the differences between Ts’s and Tv’s on regulatory activity are conservative for several reasons. We did not limit our analyses to mutations or variants that influence regulatory activity, nor did we restrict our analysis to binding sites for TFs that are more sensitive to Tv’s. We also did not remove from our analysis any regulatory elements with low activity. Our highly inclusive and conservative approach is important to demonstrate that there is a differential overall effect of Tv’s and Ts’s on regulatory element activity, but leaves to future studies a detailed understanding of the specific circumstances when Tv’s disproportionately alter that activity. We expect that elucidating those circumstances will improve prediction of the effects of noncoding variants on both molecular and organismal phenotypes.

Several mechanisms may explain the stronger effects of Tv’s. TFs may recognize the purine or pyrimidine structure rather than the specific nucleotide or, alternatively, Tv’s may disproportionately alter the DNA backbone, impacting the binding of TFs that recognize backbone shape [39, 40]. One demonstration of that principle comes from the Hox family of TFs that bind DNA by recognizing both sequence and shape independently of each other [25]. Many previous analyses have also suggested that TFs may bind through both sequence direct recognition and indirect recognition [41–45]. Direct recognition occurs when a protein interacts directly with the amino acid sequence of the DNA. Conversely, indirect recognition occurs when proteins interact with the DNA structure. Moreover, some groups have shown that including DNA structure and physiochemical features significantly improves predictions of TF binding across the genome [41, 44]. The importance of DNA shape and structure corroborates the results presented in our study, and provides a potential mechanism explaining why Tv’s have larger impacts on TF binding and regulatory activity than Ts’s. Understanding those principles of TF recognition may further inform whether specific classes of TFs are particularly impacted by Tv’s.

## Conclusions

In this work, we demonstrate that transversions have a greater impact on regulatory element activity than transitions. A likely mechanism is that transversions alter the minor groove width and roll of DNA more than transitions, leading to a greater impact on TF binding. These findings provide new insights into the ways that different types of genetic variation can have distinct effects on gene regulation, and suggests that considering whether a variant is a transversion or a transition may be valuable for studying the genetics of gene regulation in many contexts.

## Methods

### Predicted effects of mutations on DNA shape parameters

To estimate the effects of Ts’s and Tv’s on DNA shape parameters, we generated a set of random DNA sequences that differed by a single nucleotide, and then used a computational model to predict DNA shape for each sequence. Specifically, we generated 100,000 random 503 bp DNA sequences. We then converted the middle nucleotide in each sequence to all other possible nucleotides, thus yielding 400,000 sequences. We then predicted the minor groove width, roll, propeller twist, and helical twist across each sequence using DNAshapeR [26]. To estimate the effect of Ts’s and Tv’s on those shape parameters, we summed the absolute difference in each parameter between pairs of sequences that differed by a single Ts or Tv, respectively. We compared the effect of Ts’s and Tv’s on each parameter using a linear regression model that included the identity of the starting sequence as a covariate.

### Predicted effects of mutations on TF binding

The set of all non-redundant TF binding position frequency matrices (PFMs) were retrieved from the JASPAR database [28]. For each PFM, a pseudocount of 0.1 was added to every element, and the PFM was converted to a position specific scoring matrix (PSSM). The most likely (i.e. consensus) binding sequence was determined for each PSSM. We defined the PSSM score for a given DNA sequence as the sum of the corresponding positions in the PSSM. Then, for each position in each consensus sequence, we calculated the PSSM score of having every possible nucleotide at that position and, subsequently, the change in PSSM score when mutating any nucleotide to any other nucleotide at that position. The final values along with covariates such as the JASPAR motif ID and the position in the motif data were output as a table that was used for statistical analysis. PSSM generation and mutation scoring was performed using BioPython libraries, and statistical analysis was performed in R.

### Effects of transitions and transversions in saturation mutagenesis (Patwardhan et al.) dataset

Data from saturation mutagenesis of three regulatory elements were collected [19] and reformatted to give the effect of every possible mutation at every position assayed. Effects from replicate experiments were averaged. A series of linear regression models was then used to evaluate the effect of Ts’s and Tv’s on regulatory element activity while accounting for differences in regulatory element activity between elements and location of the mutation within each element. The specific models used along with coefficients and test statistics are provided in Additional file 1: Table S7. All analysis was performed using R.

### Allele specific binding analysis

We analyzed two publicly available allele-specific binding datasets, one based on the binding of multiple 42 TFs to the diploid personal genome sequence of NA12878, the other based on the binding of CTCF to SNPs discovered through ChIP-seq in 6 different LCLs [30]. We computed the Tv frequency in allele-specific variants and in all variants tested and compared the two frequencies by transforming the assumed binomial distribution to a standard normal and performing a two-tailed Z-test. We pooled allele-specific variants across TFs or across cell lines, making sure to collapse redundant variants. We were ignorant of the overlap of all tested SNPs across cell lines and for simplicity used the mean number of SNPs tested as the null model sample size for that dataset.

### Custom amplicon design and capture

Custom amplicon design and capture were performed as described in Vockley, Guo, & Majoros et al. [37] with the following difference: The number of individuals was increased from 95 to 760.

### Variant calling and phasing

Variant calling and phasing was performed as described in Vockley, Guo, & Majoros et al. [37] with the following difference: The number of individuals was increased from 95 to 760.

### POP-STARR-seq

Population STARR-seq libraries and haplotype effect size calculations were conducted as previously published by Vockley, Guo, & Majoros et al. [37]. In total, we captured 104 DHSs from the genomes of 760 donors via multiplex PCR (Additional files 2, 5 and 6: Tables S1-S3). We sequenced the captured DNA, and called variants using the Genome Analysis Toolkit (GATK) according to Best Practices recommendations [46–48] (Additional file 7: Table S4). The custom amplicon libraries were combined into eight pools (95 individuals per pool) in equimolar ratios. These pools were then amplified and cloned into the STARR-seq backbone. Each pool was transformed into Stellar chemically competent cells per manufacturer protocol. Transformations were recovered for 1 h in SOC medium while shaking (225 rpm at 37 °C) and then incubated for 16 h in 250 mL of LB while shaking (225 rpm at 37 °C). The resulting plasmid reporter input libraries were isolated using a MaxiPrep Kit (Promega). The 8 purified libraries were then pooled in equimolar ratios to create a single plasmid input library. This library was then transfected into T-175 flasks containing HepG2 cells at ~70% confluency with Fugene HD (Promega) at a 5.5:1 ratio of Fugene:DNA. In total, 3 replicate transfections were performed. RNA was harvested after ~48 h Primer sequences for library construction are included in Additional file 1: Table S8.

### Comparing effects of Ts’s and Tv’s on regulatory element activity

To determine the number of Ts’s and Tv’s between haplotypes, we grouped haplotypes by amplicon. This ensured that each haplotype was compared to only those with the exact same length and start/stop coordinates. For each amplicon, we designated one haplotype at random as the “reference haplotype”. For each group of haplotypes, we counted the number of Ts’s and Tv’s that differed between each haplotype within the group and the reference haplotype. The change in effect magnitudes between the haplotypes in each amplicon group were calculated as follows: ${\left|\right| \log}_2\left( effect\; size\; of\; haplotype\right)$| $-$ |${ \log}_2\left( effect\; size\; of\; reference\; haplotype\right)$||

Amplicon groups which did not contain at least one haplotype with an effect size *p*-value < 0.05 were excluded from the analysis. Linear regressions were performed using the lm() function in R. When performing regressions we included amplicon number as a variable.

## Additional files

Additional file 1: Figure S1. and **Tables S7.** and **S8. Figure S1.** Amplicons targeting DHS and active histone markers in multiple cell lines. In total, 104 DHS were captured using 174 amplicons. Amplicons were tiled across target regions and also captured at least 50 bp upstream and downstream of each DHS. Amplicon are ~400-425 bp in length. **Table S7.** Effects of Tv’s on regulatory element activity in Patwardhan et al. dataset. **Table S8.** Population STARR-seq primer sequences. (DOCX 255 kb)

Additional file 2: Table S1. DHS coordinates at 3q25 (BED format). (XLSX 11 kb)

Additional file 3: Table S5. Haplotype Sequences (FASTA format). (XLSX 154 kb)

Additional file 4: Table S6. Haplotype Effects. (XLSX 64 kb)

Additional file 5: Table S2. Custom Amplicon Coordinates (BED format). (XLSX 12 kb)

Additional file 6: Table S3. Custom Amplicon Probe Sequences (BED format). (XLSX 21 kb)

Additional file 7: Table S4. Variant Calls in 760 Individuals (VCF format). (XLSX 2056 kb)

## Abbreviations

DHS: DNase-Hypersensitive site
GATK: Genome analysis toolkit
HelT: Helical twist
LCL: Lymphoblastoid cell line
MGW: Minor groove width
POP-STARR: Population-scale STARR-seq
ProT: Propeller twist
PWM: Position weight matrix
SNP: Single nucleotide polymorphism
STARR-seq: Self-transcribing active regulatory region sequencing
TF: Transcription factor
Ts: transition
Tv: transversion
UTR: Untranslated region
