# Supplementary material for: Transversions have larger regulatory effects than transitions
Source: BMC Genomics. 2017 May 19;18:394. doi: 10.1186/s12864-017-3785-4 (PMC5438547; doi:10.1186/s12864-017-3785-4)
Supplement: Supplementary file 1 — and Tables S7. and S8. Figure S1. Amplicons targeting DHS and active histone markers in multiple cell lines. In total, 104 DHS were captured using 174 amplicons. Amplicons were tiled across target regions and also captured at least 50 bp upstream and downstream of each DHS. Amplicon are ~400-425 bp in length. Table S7. Effects of Tv’s on regulatory element activity in Patwardhan et al. dataset. Table S8. Population STARR-seq primer sequences. (DOCX 255 kb) [file 12864_2017_3785_MOESM1_ESM.docx]

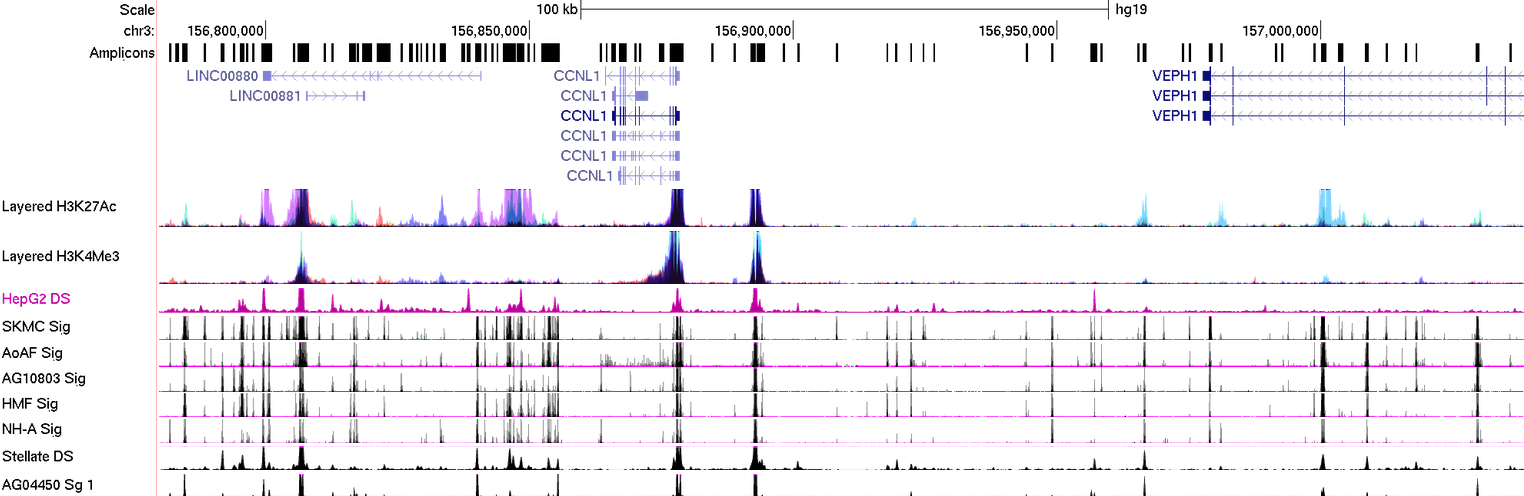


**Figure S1.** Amplicons targeting DHS and active histone markers in multiple cell lines. In total, 104 DHS were captured using 174 amplicons. Amplicons were tiled across target regions and also captured at least 50 bp upstream and downstream of each DHS. Amplicon are ~400-425 bp in length.

**Table S7:** Effects of Tv’s on regulatory element activity in Patwardhan *et al.* dataset

| Model | β | se | t | Pr(>t) |
| --- | --- | --- | --- | --- |
| effect ~ tstv | 0.015 | 0.007 | 2.069 | 0.019 |
| effect ~ tstv * enhancer | 0.031 | 0.014 | 2.185 | 0.014 |
| effect ~ tstv * enhancer * distance_from_element_center | 0.071 | 0.027 | 2.616 | 0.004 |

**Table S8:** Population STARR-seq Primer Sequences

| TS2SSF: | TAGAGCATGCACCGGACACTCTTTCCCTACACGACGCTCTTCCGATCT |
| --- | --- |
| TS2SSCIDRR: | GGCCGAATTCGTCGAGTGACTGGAGTTCAGACGTGTGCTCTTCCGATCT |
| CIDRread2 | GTGACTGGAGTTCAGACGTGTGCTCTTCCGATCT |
| SSRT | CAAACTCATCAATGTATCTTATCATG |
| SS-spliced F | GGGCCAGCTGTTGGGGTGTCCAC |
| SS-spliced R | CTTATCATGTCTGCTCGAAGC |
| CIDRBCread | AGATCGGAAGAGCACACGTCTGAACTCCAGTCAC |
| CIDRBC1 | CAAGCAGAAGACGGCATACGAGATCGTGATGTGACTGGAGTTCAGACGTGTGCTCTTCCGATCT |
| CIDRBC2 | CAAGCAGAAGACGGCATACGAGATACATCGGTGACTGGAGTTCAGACGTGTGCTCTTCCGATCT |
| CIDRBC3 | CAAGCAGAAGACGGCATACGAGATGCCTAAGTGACTGGAGTTCAGACGTGTGCTCTTCCGATCT |
| CIDRBC4 | CAAGCAGAAGACGGCATACGAGATTGGTCAGTGACTGGAGTTCAGACGTGTGCTCTTCCGATCT |
| CIDRBC5 | CAAGCAGAAGACGGCATACGAGATCACTGTGTGACTGGAGTTCAGACGTGTGCTCTTCCGATCT |
| CIDRBC6 | CAAGCAGAAGACGGCATACGAGATATTGGCGTGACTGGAGTTCAGACGTGTGCTCTTCCGATCT |
| CIDRBC7 | CAAGCAGAAGACGGCATACGAGATGATCTGGTGACTGGAGTTCAGACGTGTGCTCTTCCGATCT |
| CIDRBC8 | CAAGCAGAAGACGGCATACGAGATTCAAGTGTGACTGGAGTTCAGACGTGTGCTCTTCCGATCT |
| CIDRBC9 | CAAGCAGAAGACGGCATACGAGATCTGATCGTGACTGGAGTTCAGACGTGTGCTCTTCCGATCT |
| CIDRBC10 | CAAGCAGAAGACGGCATACGAGATAAGCTAGTGACTGGAGTTCAGACGTGTGCTCTTCCGATCT |
| CIDRBC11 | CAAGCAGAAGACGGCATACGAGATGTAGCCGTGACTGGAGTTCAGACGTGTGCTCTTCCGATCT |
